# Primary Leiomyosarcoma of the Orbit and Conjunctiva: A Report of Four Cases With a Review of the Literature

**DOI:** 10.7759/cureus.59297

**Published:** 2024-04-29

**Authors:** Shebin Salim, Kirthi Koka, Soham Pal, Nisar S Poonam, Bipasha Mukherjee

**Affiliations:** 1 Orbit, Oculoplasty, Reconstructive and Aesthetic Services, Sankara Nethralaya, Chennai, IND

**Keywords:** leiomyosarcoma, orbit, conjunctiva, smooth muscle tumor, soft tissue sarcoma

## Abstract

Leiomyosarcomas (LMS) are common soft tissue tumors in the body. Primary orbital and conjunctival LMS are, however, rare. Herein, we describe the diverse clinical presentations, histopathological features, and management outcomes of three cases of primary LMS of the conjunctiva and one case of primary orbital LMS. The first patient was a 40-year-old female with primary orbital LMS who developed recurrence following wide local excision. The remaining three cases were primary conjunctival LMS. All four patients underwent orbital exenteration and were disease-free at a mean follow-up period of 18.64 months. LMS is known for local recurrences and metastasis. Complete surgical excision and prompt adjuvant radiotherapy can improve the prognosis.

## Introduction

Leiomyosarcomas (LMS) are the most common soft tissue sarcomas in the body. Nevertheless, primary LMS of the orbit and the conjunctiva are extremely rare. To date, merely 10 cases of primary orbital and 12 cases of primary conjunctival LMS have been reported in the literature [1-20]. There have been reports of metastatic and/or secondary orbital LMS following radiation therapy for retinoblastoma or due to direct extension from adjacent paranasal sinuses [6]. We herein report three cases of primary conjunctival LMS and one case of primary orbital LMS with a review of the published literature.

## Case presentation

Case 1

**Figure 1:**
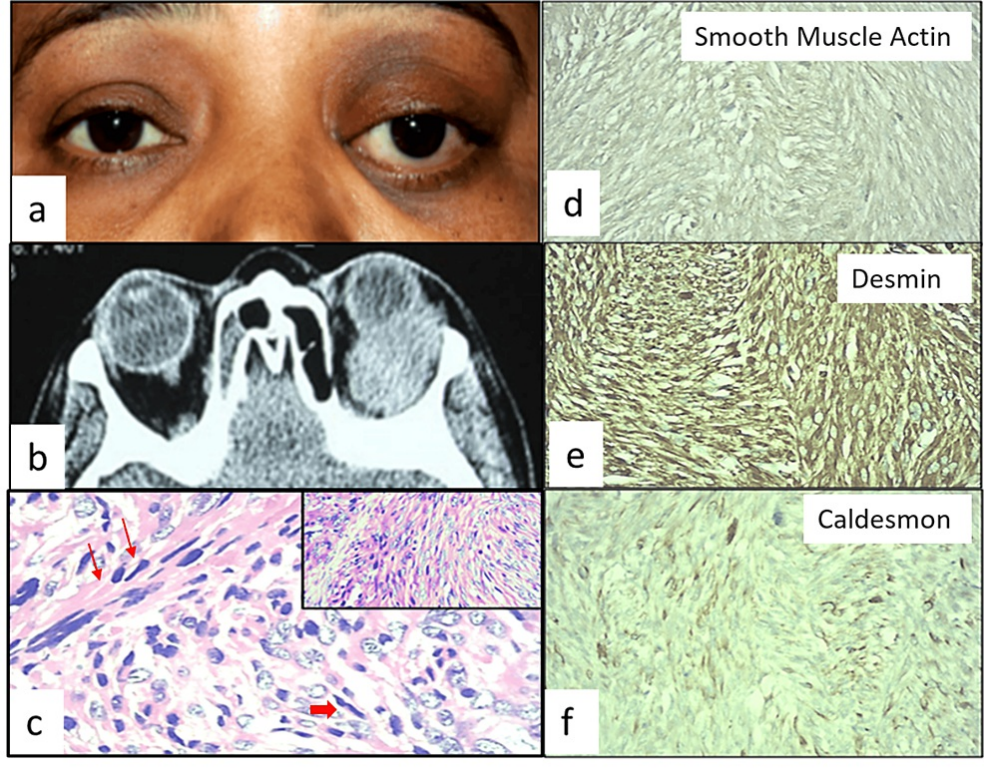
Case 1 (a) External color photograph of a 40-year-old female showing left eye proptosis with inferomedial dystopia. (b) CT orbit axial view showing a homogeneous solid soft tissue lesion in the superotemporal quadrant of the left orbit enveloping the globe. (c) Histopathology showing spindle cell (thin arrow) tumor with severe atypia and fascicular pattern. Tumor cells have elongated nuclei with blunt ends (thick arrows) (stain: eosin and hematoxylin, magnification: 400×). Inset showing (low magnification: 40×) fascicular pattern. Immunohistochemistry shows positivity for (d) SMA, (e) desmin, and (f) caldesmon; all three are cytoplasmic stains that are shown by the brown-colored cells. CT: computed tomography, SMA: smooth muscle actin

A 40-year-old Asian-Indian female presented with complaints of rapidly progressive, painless prominence of her left eye for the last three months. On examination, her best-corrected visual acuity was 6/6, N6 in both eyes. External examination showed left eye abaxial proptosis of 4 mm recorded on Hertel's exophthalmometry with 2 mm inferomedial dystopia and minimal restriction of ocular movements (Figure 1a). The rest of the anterior and posterior segment examinations were within normal limits. Computed tomography (CT) scan of orbits showed a homogeneous soft tissue lesion in the superotemporal quadrant of the left eye enveloping the globe, suggestive of a lymphoproliferative lesion. The lacrimal gland could not be identified separately (Figure 1b).

The patient underwent an excisional biopsy through a lateral orbitotomy approach. Histopathology revealed a cellular spindle cell tumor, infiltrating the orbit with partial capsulation. The spindle cells showed severe atypia and a fascicular pattern of arrangement. Areas of necrosis were present, and the tumor cells had elongated nuclei with blunt ends. Mitosis of 20-25 per high-power fields was noted, suggestive of high-grade malignant LMS (Figure 1c). Immunohistochemistry (IHC) was positive for smooth muscle actin (SMA), desmin, and caldesmon (Figure 1d-1f) and negative for S100 and CD34, thus confirming the diagnosis of LMS. A full-body positron emission tomography (PET) scan done postoperatively showed metabolically active soft tissue in the superolateral aspect of the left orbit and no evidence of primary elsewhere or any systemic metastasis.

We, following a multidisciplinary tumor board meeting, discussed the options of exenteration vis-a-vis adjuvant radiation with the patient. An attempt to salvage the globe was made as per the patient's wishes. The patient was advised to undergo adjuvant external beam radiotherapy (EBRT) three weeks after surgery. However, the patient did not return on time ostensibly due to financial constraints. She received 60 Gy of radiation in 30 divided fractions after three months of surgery. A magnetic resonance imaging (MRI) done 10 months after excision showed an ill-defined lobulated, infiltrating, intraorbital mass lesion along the superolateral aspect of the left orbit with mild diffusion restriction and suggestive of a recurrence. She underwent left orbital exenteration elsewhere, and the histopathology examination confirmed a recurrent LMS. MRI scan done two months following exenteration showed no evidence of any residual or recurrent lesion. The patient is currently under review and is disease-free at three years follow-up.

Case 2

**Figure 2:**
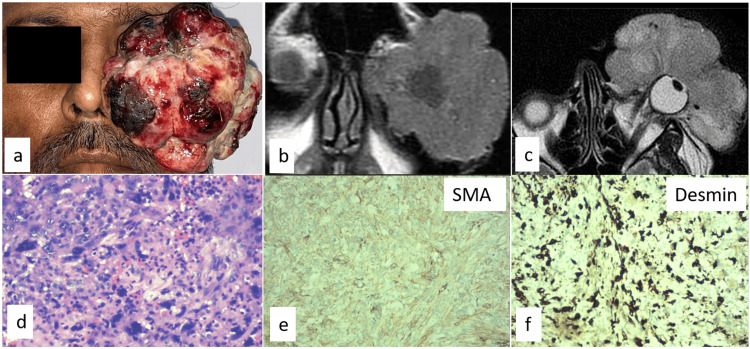
Case 2 (a) External color photograph of a 50-year-old male showing a fungating tumor over the left eye. MRI (b) coronal and (c) axial cuts showing large exophytic lesion enveloping the ocular surface, which is isointense in T1, and intermediate signal in T2-weighted images. (d) Histopathology showing tumor composed of malignant spindle cells and pleomorphic cells with severe atypia and mitosis (stain: eosin and hematoxylin, magnification: 400×). Immunohistochemistry showing positivity for (e) SMA and (f) desmin. MRI: magnetic resonance imaging, SMA: smooth muscle actin

A 50-year-old Asian-Indian male presented with a painless, slow-growing mass in the left eye for two years. On examination, a large fungating cauliflower-shaped mass was present over the surface of the left eye, which bled on touch (Figure 2a). The visual acuity was 6/6, N6 in the right eye and no perception of light in the left eye. A magnetic resonance imaging (MRI) of the orbit showed a large exophytic lesion enveloping the ocular surface, which was isointense in T1, and intermediate signal in T2 (Figure 2b, 2c). The clinical and imaging features were suggestive of ocular surface neoplasia.

An incisional biopsy under frozen section control was done. Histopathology of the frozen section was suggestive of either a poorly differentiated carcinoma or an undifferentiated pleomorphic sarcoma (Figure 2d). Hence, he underwent an eyelid-sparing exenteration under general anesthesia, considering the extensive involvement of the tumor and the high grade on histopathology. Histopathology revealed the tumor to be arising from the conjunctiva with orbital and scleral invasion but without any intraocular extension. Areas of necrosis were seen. The Ki-67 was 36%. Immunohistochemistry showed positive smooth muscle actin and desmin (Figure 2e, 2f) and negative S100 and CD34, which was suggestive of conjunctival LMS. PET-CT done one month postoperatively showed minimal soft tissue with mild metabolic activity in the posterior orbit with no evidence of disease elsewhere, thus confirming a diagnosis of primary conjunctival LMS. The patient received adjuvant EBRT of 60 Gy in 30 fractions, eight weeks post-surgery. At 18 months follow-up, there was no evidence of local recurrence or systemic spread.

Case 3

A 73-year-old Asian-Indian male presented with complaints of right eye prominence and growth for the past eight months. He was hypertensive with chronic kidney disease and had undergone a renal transplant one year back. CT scan of the orbit done elsewhere showed an ill-defined isodense lesion in the right lateral orbit infiltrating the lateral rectus muscle. An incisional biopsy was performed two months back, which was suggestive of either an undifferentiated pleomorphic sarcoma or an LMS.

**Figure 3:**
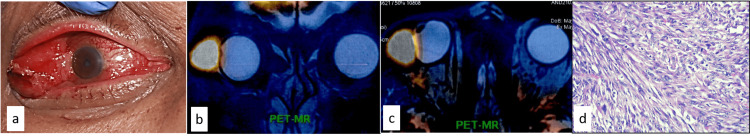
Case 3 (a) External color photograph of a 73-year-old male showing a reddish mass in the superotemporal quadrant of the right eye. PET-CT with MRI of the orbit of the same patient (b) coronal and (c) axial cuts showing the metabolically active lesion in the superotemporal quadrant indenting the globe. (d) Histopathology showing malignant spindle cell tumor showing fascicular pattern and nuclear atypia (stain: eosin and hematoxylin, magnification: 400×). PET-CT: positron emission tomography-computed tomography, MRI: magnetic resonance imaging

On presentation at our clinic, his best-corrected visual acuity was 3/60, N6 in the right and 6/6, N6 in the left eye. External examination showed a reddish-pink, non-tender mass in the superotemporal quadrant of the right eye, which was adherent to the globe and bled on touch (Figure 3a). A PET-CT with magnetic resonance hybrid fusion showed a metabolically active heterogeneously enhancing lesion infiltrating the lacrimal gland, lateral rectus and superior rectus muscle, and lateral wall of the globe with globe indentation (Figure 3b, 3c). There was no evidence of nodal or distant metastasis. The lesion showed an increase in size compared to the previous CT scan. On account of the highly malignant nature, the patient underwent a right orbital lid-sparing exenteration under general anesthesia. Histopathology showed a malignant spindle cell tumor with a storiform pattern and nuclear atypia (Figure 3d). The spindle cells showed a cigar-shaped nucleus with cytoplasmic vacuoles. The mitosis varied from 16 to 25 mitosis per 10 high-power field. No areas of necrosis were seen. IHC showed positivity for SMA, vimentin, S100, and muscle-specific actin (MSA), confirming the diagnosis of LMS. An immunohistochemistry marker, Epstein-Barr encoding region in situ hybridization (EBER-ISH), was done to look for any Epstein-Barr virus-related infection, which was found to be negative. He received adjuvant EBRT of 54 Gy in 27 divided fractions six weeks post-surgery. At 10 months follow-up, the patient had no local recurrence or systemic spread clinically and was referred for an exenteration prosthesis.

Case 4

**Figure 4:**
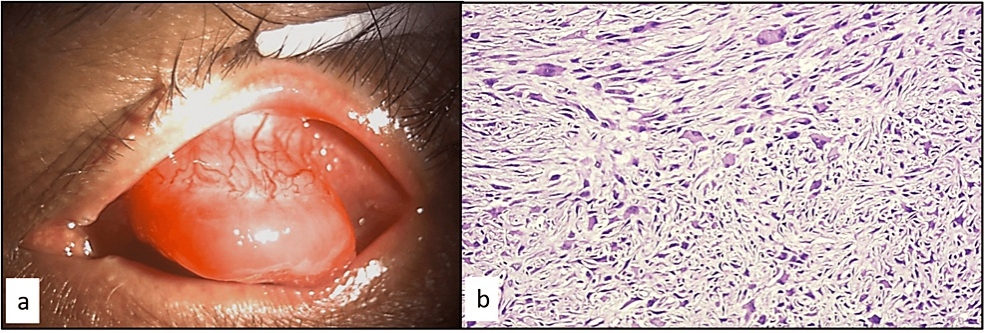
Case 4 (a) External color photograph of a 72-year-old male showing a pedunculated, fleshy, pinkish mass in the superotemporal conjunctiva. (b) Histopathology showing malignant spindle cell tumor with severe atypia and fascicular pattern (stain: eosin and hematoxylin, magnification: 400×).

A 72-year-old Asian-Indian male presented with complaints of recurrent growth in his left eye for 15 days duration following an excisional biopsy done elsewhere two months back. The growth was present for one year before the procedure. The histopathology was suggestive of LMS. His best-corrected visual acuity was 6/6, N6 in the right eye and 6/12, N12 in the left. External examination revealed a fleshy, pedunculated, pinkish mass adherent to the sclera and extending up to the limbus in the superotemporal conjunctiva (Figure 4a). Ocular movements revealed restricted elevation and abduction. Scleral invasion was noted on MRI of the orbit. In view of the early recurrence and scleral invasion, the patient underwent left eye exenteration. Histopathology showed a malignant spindle cell tumor with a fascicular pattern having severe atypia and abnormal mitosis (Figure 4b). IHC was positive for SMA and calponin, suggestive of LMS. The Ki-67 was 30%-40%. A PET-CT showed no evidence of systemic metastasis. The patient was advised adjuvant EBRT of 60 Gy in 30 fractions over six weeks. Since he wanted to continue further treatment in his hometown, he was advised to review two months after completing radiation therapy.

## Discussion

LMS is one of the most frequent soft tissue sarcomas with an incidence ranging between 10% and 20% of all newly diagnosed cases of sarcomas [14]. It is more common in the older population. Common locations include the abdomen, retroperitoneum, larger blood vessels, and uterus. LMS of the orbit or the conjunctiva is rare; only 22 cases have been reported in the literature to date (Table 1).

**Table 1:** Review of literature on cases of primary orbital and conjunctival leiomyosarcoma M: male, F: female, NA: not available, CT: computed tomography, MRI: magnetic resonance imaging, SMA: smooth muscle actin, CK: cytokeratin, MSA: muscle-specific actin

Serial number	Author	Age (years)/sex	Location	Presentation	Imaging features	Management	IHC positive for	Follow-up
1	Jakobiec et al. (1975) [1]	58/F	Orbit	Blurring of vision, proptosis (15 months)	X-ray: symmetrical enlargement of the right orbit	Excision	NA	12 months: no recurrence, 15 months: died due to metastasis
		59/F	Orbit	Proptosis (18 months)	NA	Excision:4 times, exenteration, radiotherapy	NA	67 months: multiple local recurrences, died due to lung and brain metastasis
2	De Wolff-Rouendaal (1976) [2]	20/F	Conjunctiva	Limbal mass (6 months), case of xeroderma pigmentosa	NA	Incisional biopsy, exenteration	NA	6 months: died due to unrelated cause
3	Wojno et al. (1983) [3]	36/M	Orbit	Medial orbital mass (6 months)	CT: lesion suggestive of small fibroma	Excision	NA	NA
4	Meekins et al. (1988) [4]	82/F	Orbit	Proptosis and diminution of vision (5 weeks), diplopia (4 weeks)	CT: homogeneous lateral orbital mass with retrobulbar extension	Excision, radiotherapy	NA	5 months: died due to stroke
5	White et al. (1991) [5]	66/M	Conjunctiva	Pseudopterygia	NA	Subtotal exenteration	Vimentin MSA	24 months: no recurrence
6	Wiechens et al. (1999) [6]	84/F	Orbit	Dystopia, mass lesion	CT: ill-defined mass infiltrating the entire orbit	Exenteration, radiotherapy	NA	3 months: recurrence, 14 months: died due to systemic cause
7	Hou et al. (2003) [7]	56/F	Orbit	Diplopia, proptosis (10 months)	MRI: extraconal mass with sinus extension	Excision, chemotherapy (doxorubicin and ifosfamide)	SMA, desmin	2 months: suspected recurrence
8	Lin et al. (2005) [8]	84/F	Orbit	Lid nodule (1 month)	CT: medial extraconal mass	Excision, radiotherapy	SMA	3 years: no recurrence
9	Yoon et al. (2006) [9]	59/M	Conjunctiva	Limbal mass (2 months)	NA	Excision	Vimentin, SMA, desmin, CD68	3 months
10	Yeniad et al. (2009) [10]	79/F	Orbit	Proptosis (6 months)	MRI: heterogeneous, well-defined mass with marked heterogeneous contrast enhancement	Excision	SMA	12 months: no recurrence
11	Guerriero et al. (2011) [11]	56/F	Conjunctiva	Conjunctival mass (4 months)	CT of the orbit: conjunctival exophytic lesion with orbital extension, CT of the body: metastatic lymphadenopathy	Exenteration	Actin, vimentin	NA
12	Kenawy et al. (2012) [12]	37/F	Conjunctiva	Conjunctival mass (8 weeks)	CT: enhancing soft tissue mass at the insertion of the medial rectus	Excision	SMA, vimentin	12 months: no recurrence
13	Wan-Wei et al. (2015) [13]	60/F	Conjunctiva	Limbal mass (2 months)	NA	Excision	Actin, vimentin, S100	5 months: no recurrence
14	Nair et al. (2015) [14]	34/M	Conjunctiva	Conjunctival mass (2 years)	CT: hyperdense lesion extending from the ocular surface to the anterior orbit	Exenteration	SMA, vimentin	12 months: no recurrence
		39/M	Conjunctiva	Conjunctival mass (4 months)	NA	Excision, ruthenium plaque-brachytherapy	SMA, vimentin	14 months: no recurrence
15	Montes et al. (2017) [15]	81/M	Conjunctiva	Conjunctival mass (2 years)	CT: soft tissue density on the ocular surface	Evisceration, radiotherapy	SMA, vimentin	NA
16	Wadee (2017) [16]	38/M	Conjunctiva	Conjunctival growth (duration not clear)	NA	Excision with cryotherapy to base	Desmin, SMA, MSA, H-caldesmon	NA
17	Zhou et al. (2017) [17]	32/F	Orbit	Proptosis, diplopia	MRI: solid mass in the left orbit with invasion to the adjacent tissue	Incisional biopsy: further treatment details not available	SMA, vimentin	NA
18	Chaugule et al. (2018) [18]	71/M	Orbit	Proptosis (4 months)	CT: well-defined, homogeneous, iso-hyperdense lesion in the lateral extraconal compartment	Exenteration	SMA, calponin	5 months: no recurrence
19	De Groot et al. (2019) [19]	79/M	Conjunctiva	Conjunctival mass (6 months)	NA	Excision twice, strontium-90 brachytherapy	SMA	6 months: recurrence, 4 years: died due to unrelated disease
20	Gameiro Filho et al. (2022) [20]	45/M	Conjunctiva	Recurrent conjunctival mass	NA	Extended enucleation	Vimentin, calponin, desmin, caldesmon, CD68, SMA	30 months: no recurrence
21	Present study (2022)	40/F	Orbit	Proptosis (3 months)	CT: homogeneous solid soft tissue lesion in the superotemporal quadrant enveloping the globe	Excision, radiotherapy, exenteration	SMA, desmin, caldesmon, CK (AE1/AE3)	36 months: no recurrence
		50/M	Conjunctiva	Fungating mass (2 years)	MRI: exophytic lesion, enveloping the ocular surface	Extended enucleation, radiotherapy	SMA, vimentin, desmin	18 months: no recurrence
		73/M	Conjunctiva	Proptosis, decreased vision (8 months)	CT: ill-defined isodense lesion in the right lateral orbit infiltrating the lateral rectus muscle	Exenteration, radiotherapy	SMA, vimentin	10 months: no recurrence
		72/M	Conjunctiva	Recurrent growth post-excisional biopsy (15 days)	MRI: conjunctival mass with scleral invasion	Exenteration, radiotherapy	SMA, calponin	NA

Primary orbital LMS are usually present in older patients with a female predominance. Only two cases have been reported in males [3,18]. On the contrary, primary conjunctival LMS are more commonly reported in males. These tumors have been postulated to arise from smooth muscle in the blood vessel wall, Muller's muscles, orbital septal fascia, or de novo from smooth muscle precursor cells [1,3]. A review of the literature on small conjunctival LMS shows a predilection for the limbus. De Groot et al. [19] speculate a possible origin from the pluripotent limbal stem cells. Epstein-Barr virus infection in the setting of severe immunosuppression and post-radiotherapy in young children have been considered possible predisposing factors [6,19]. Our third patient had a history of renal transplantation and was on immunosuppressants. Nevertheless, the EBER-ISH technique to detect the presence of Epstein-Barr virus infection was negative.

Since orbital LMS are rare, they pose a diagnostic and therapeutic challenge. The common presenting features of orbital LMS are painless proptosis associated with diplopia and vision loss, while conjunctival LMS usually presents with conjunctival growth mimicking ocular surface neoplasia [14,16,19]. Guerriero et al. [11] reported a case similar to our second case where the conjunctival LMS presented as a large exophytic ulcerative mass. The delay in presentation in our second case was probably due to a poor socioeconomic background. While our first case with orbital LMS presented with a fast-growing mass of three months duration, the conjunctival LMS cases had a relatively slower course ranging from eight months to two years. Nevertheless, all four cases eventually required exenteration irrespective of the duration. It is also possible that considering the orbital location, there was a delay in the appearance of symptoms in the first case. With the small number of patients in this series, we cannot say if the growth rate of the tumor has any prognostic implications.

MRI and CT have been used as imaging tools for the evaluation of LMS [7, 17,18]. The presence of hypointensity on T2-weighted images with a peripheral rim of contrast enhancement can aid in diagnosis as suggested by Hou et al. [7]. Radiographically, our first case appeared to be of lymphoproliferative etiology on CT scan due to its homogeneous appearance and characteristic molding around the globe. The presentation in our second case simulated an advanced case of ocular surface neoplasia.

Before the era of immunohistochemistry, LMS were identified based on finding characteristic smooth muscle features on light and electron microscopy [3]. The histopathological features described in the literature include hypercellular interlacing fascicles of large spindle cells with few round cells, centrally located cigar-shaped nucleus, nuclear atypia, a high mitotic rate, and coagulative tumor cell necrosis [6,10,18]. Immunohistochemistry helps in differentiating LMS from peripheral nerve sheath tumors and confirms its myogenic origin. LMS are mostly reactive for alpha-smooth muscle actin, desmin, and h-caldesmon on immunohistochemistry and are usually negative for S100 and human melanoma black-45 (HMB-45) [18]. The other differential diagnosis includes very rare tumors of mesenchymal origin including solitary fibrous tumor (SFT), synovial sarcoma, and spindle cell rhabdomyosarcoma. SFT characteristically shows a haphazard arrangement of spindle cells on histopathology. The presence of the *NAB2-STAT6* gene fusion is now the hallmark of the diagnosis of SFT. Synovial sarcoma of the orbit is an extremely rare tumor that usually presents as a well-defined lesion with calcification. More than 90% of these tumors show *SYT-SSX* gene fusion. Spindle cell rhabdomyosarcoma of the orbit is an uncommon variant and has a predilection for young males. They show histopathology similar to LMS. IHC also shows overlapping features, but positivity for myogenin is used as a standard approach to diagnosis, as myogenin shows no reactivity in cases of LMS.

LMS are known for their propensity for hematogeneous spread and local recurrence. Our fourth case had a recurrence as early as 15 days following local excision. A thorough systemic evaluation is mandatory to rule out primaries elsewhere and systemic metastasis. Orbital exenteration with the removal of any involved bone was considered the treatment of choice for primary orbital LMS [1]. However, some favor wide surgical excision of tumors with negative margins when possible [10]. In the case of limited ocular surface tumors with intraocular extension, wide local excision with enucleation is preferred over evisceration. Wojno et al. [3] performed wide surgical excision with negative margins, but long-term follow-up was not clarified in their study.

Meekins et al. [4] and Lin et al. [8] reported the use of adjuvant radiotherapy of 50 Gy delivered in 25 fractions even after complete excision of the tumor. Long-term follow-up was not available in the case reported by Meekins et al. [4] as the patient died after five months due to stroke, whereas Lin et al. [8] reported no evidence of disease at three years follow-up. Adjuvant radiotherapy is also recommended following a recurrence of the tumor along with palliative exenteration [6]. Although the literature shows favorable outcomes with globe preservation in most cases, the delay in receiving adjuvant radiotherapy could have led to the local recurrence, thus necessitating an exenteration in our first case. Regarding our fourth case, whether complete surgical excision with margin clearance was achieved in primary surgery was not clear. Also, the patient did not receive any adjuvant radiation post-procedure. In cases of conjunctival LMS, for localized lesions that can be removed without causing functional loss, a globe-sparing complete excision can be considered, while exenteration is done in cases with orbital extension since margin clearance cannot be achieved in case of orbital involvement. Adjunctive brachytherapy to the base can be done following globe-sparing excision if margins are not clear [14,19]. The orbit being a confined space, tumor-free margins may not be achieved in all cases, and we cannot exclude the possibility of microscopic residues in these cases, thus necessitating the need for adjuvant radiotherapy treatment even after exenteration. Radiotherapy helps in local disease control and decreases the local recurrence rate.

The role of chemotherapy in orbital LMS is controversial with few reports and is most often reserved for metastatic tumors. Doxorubicin is the chemotherapeutic agent of choice for systemic chemotherapy. Hou et al. [7] reported a decrease in the size of the recurrent lesion with doxorubicin and ifosfamide. Trials are being conducted on the efficacy of gemcitabine, docetaxel, and targeted therapy with pazopanib, an oral tyrosine kinase inhibitor, in cases of recurrent and metastatic LMS, which are resistant to first-line chemotherapy [19,20].

## Conclusions

It is known that primary LMS can present with diverse clinical features and imaging findings. Histopathology and immunohistochemistry are pivotal in establishing the diagnosis. Our case series reports a total of four primary LMS of the orbit and adnexa, which is the largest reported series to date. Although we cannot arrive at any definite conclusion considering the small number and short follow-up, we propose that adjuvant radiotherapy is needed in all cases with orbital involvement to prevent recurrence owing to the intrinsic aggressive behavior of this tumor. In case of rapidly progressive conjunctival lesions without any typical features of ocular surface squamous metaplasia, conjunctival LMS should be considered as a possible differential diagnosis, and an immunohistochemistry shall be done in those cases.
